# The Role of Chromosomal Instability in Cancer and Therapeutic Responses

**DOI:** 10.3390/cancers10010004

**Published:** 2017-12-28

**Authors:** Natalia Vargas-Rondón, Victoria E. Villegas, Milena Rondón-Lagos

**Affiliations:** 1 School of Biological Sciences, Universidad Pedagógica y Tecnológica de Colombia, Tunja 150003, Colombia; ingrid.vargas@uptc.edu.co; 2 Biology Program, Faculty of Natural Sciences and Mathematics, Universidad del Rosario, Bogotá 111221, Colombia

**Keywords:** chromosomal instability, therapeutic resistance, cancer outcomes, cancer prognosis, predictive markers

## Abstract

Cancer is one of the leading causes of death, and despite increased research in recent years, control of advanced-stage disease and optimal therapeutic responses remain elusive. Recent technological improvements have increased our understanding of human cancer as a heterogeneous disease. For instance, four hallmarks of cancer have recently been included, which in addition to being involved in cancer development, could be involved in therapeutic responses and resistance. One of these hallmarks is chromosome instability (CIN), a source of genetic variation in either altered chromosome number or structure. CIN has become a hot topic in recent years, not only for its implications in cancer diagnostics and prognostics, but also for its role in therapeutic responses. Chromosomal alterations are mainly used to determine genetic heterogeneity in tumors, but CIN could also reveal treatment efficacy, as many therapies are based on increasing CIN, which causes aberrant cells to undergo apoptosis. However, it should be noted that contradictory findings on the implications of CIN for the therapeutic response have been reported, with some studies associating high CIN with a better therapeutic response and others associating it with therapeutic resistance. Considering these observations, it is necessary to increase our understanding of the role CIN plays not only in tumor development, but also in therapeutic responses. This review focuses on recent studies that suggest possible mechanisms and consequences of CIN in different disease types, with a primary focus on cancer outcomes and therapeutic responses.

## 1. Introduction

Cancer is a multifactorial disease, which is characterized by the presence of a population of cells with complex and heterogeneous karyotypes [1]. Therapeutic decisions for cancer patients are primarily based on clinical and pathological parameters. In particular, tumor size, histological grade, histotype and immunohistochemical results of prognostic factors play major roles in planning therapeutic strategies [2] (e.g., targeted therapy or chemotherapy). Although this has been a successful approach, many patients relapse and/or eventually develop resistance. Despite the fact that vast technological improvements have increased our understanding of human cancers as heterogeneous diseases, current clinicopathological, immunohistochemical and molecular parameters/markers leave significant numbers of patients at risk for over- or under-treatment.

A promising therapeutic target for cancer is chromosome instability (CIN), a common feature of solid tumors. CIN has been recognized as a source of genetic variation, favoring tumor adaptations to stressful environments and cytotoxic anticancer drugs [3]. In cancer research, both numerical (aneuploidy) and structural CIN have been shown to impact carcinogenesis and possibly therapeutic responses; however, although CIN has been associated with cancer therapy, contradictory findings have been reported regarding its implications for the therapeutic response [4,5,6]. Thus, it is necessary to increase our understanding of the role CIN plays not only in tumor development but also in responses to therapy. In this review, we will discuss the impact of CIN on the prognosis of many disease types, including cancer.

## 2. CIN and Cancer

**Figure 1:**
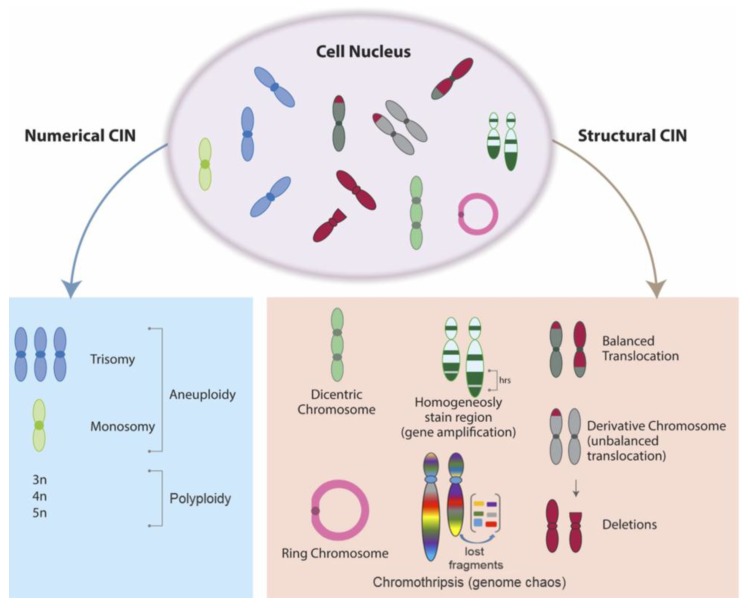
Chromosomal instability (CIN) Characteristics. CIN is characterized by aberrant distribution of chromosomes to the daughter cells deviating from the modal number (numerical CIN—aneuploidy and euploidy) or an elevated frequency of structural chromosome aberrations such as gain or loss of partial chromosomes (structural CIN).

CIN, defined as a defect that involves loss or rearrangement of the chromosomes during cell division [4], has been recognized as hallmark of cancer [7] and a source of genetic variation that favors tumor adaptations to stressful environments and cytotoxic anticancer drugs. CIN is a common feature of solid tumors and can be classified as numerical CIN or structural CIN [8]. Numerical CIN is characterized by gain or loss of whole chromosomes (aneuploidy) [3], while structural CIN is characterized by gain or loss of fractions of chromosomes [3] (Figure 1).

Aneuploidy refers to the state of abnormal chromosome numbers, which can be stable or unstable. Unstable aneuploidy may favor the simultaneous growth of various tumor subpopulations leading to inter and intratumoral genomic heterogeneity [3,9,10].

In addition, in cancers with elevated numerical and structural CIN, genome chaos has been also observed. Genome chaos defined as a process of complex, rapid genome re-organization, is characterized by the presence of extreme structural and numerical chromosomal alterations [11]. Many of these chromosomal alterations are non-recurrent abnormalities (NCCAs) and since these changes are not clonal (clonal chromosomal alterations—CCAs), they are widely ignored and therefore not reported [12].

CCAs are defined as chromosomal alterations observed at least twice within 20 to 40 randomly examined mitotic figures (range of occurrence greater than 30%) [13]. NCCAs are defined as non-recurrent chromosomal alterations observed at a frequency of less than 4% among 50–100 mitotic figures [13] and are characteristic of chaotic genomes. Examples of NCCAs include deletions, translocations, gene amplifications, inversions, chromothripsis, chromoplexy, dicentric chromosomes and duplications, among others [11,14].

Considering that CCAs are indicative of stable karyotypes and NCCAs of unstable karyotypes, it has been suggested that NCCAs are the main indicators of structural CIN and cancer evolution [15]. However, in spite of the above, NCCAs have been widely ignored, since have been considered as insignificant “noise” [16,17] and as in vitro culture artifact. Therefore, information about the presence of these alterations in many types of cancer is scarce, which limits the possibility of obtaining additional information about genomic diversity and, therefore, intra and inter tumor heterogeneity [12,17].

Taking into account that several studies have suggested that NCCAs are essential in the evolution of cancer [17,18] and, therefore can be useful in the establishment of both tumor heterogeneity and CIN [13,17], their inclusion in the study of cancer is urgent, essential and relevant.

### 2.1. Mechanisms of CIN

**Figure 2:**
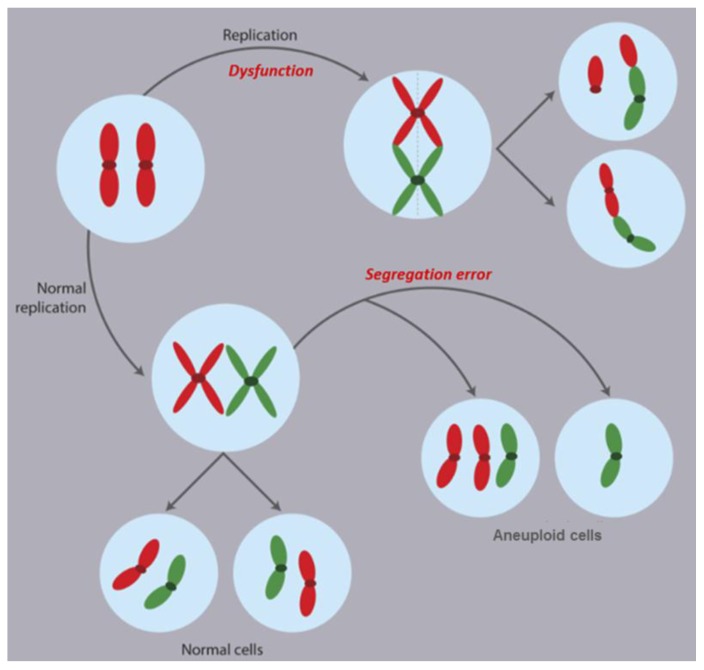
Numerical and structural CIN arise during mitotic chromosome segregation errors. Dysfunctional chromosome duplication or segregation during mitosis can conduce to whole chromosome gains and losses (numerical CIN) and/or alterations in the structure of chromosomes (structural CIN) including translocations, deletions, and derivative chromosome, among other. Both numerical and structural alterations predispose chromosomes to subsequent chromosomal alterations, thereby increasing CIN.

The mechanisms underlying CIN remain poorly understood but likely reflect dysfunctional chromosome duplication or segregation during mitosis (Figure 2). Within these mechanisms are: kinetochore-microtubule attachment errors, aberrant sister chromatid cohesion, abnormal centrosome replication, telomere attrition, and the spindle assembly checkpoint (SAC) abnormalities [19], among others. Cancer cells with CIN mis-segregate a chromosome approximately once every one to five divisions, compared with a rate of one chromosome per hundred cell divisions in stable, diploid cell lines [20,21].

During mitosis and meiosis, the SAC acts to maintain genome stability by delaying cell division until accurate chromosome segregation can be guaranteed [22], which ensures that anaphase is triggered only after all kinetochores are bound to spindle microtubules [23]. In order for chromosome segregation to be carried out with high fidelity, prior to the start of anaphase, the kinetochores must capture the microtubules of the spindle and connect the sister chromatids of each chromosome to the poles of the opposite spindle (amphitelic attachment). Once all chromosomes achieve proper bi-oriented attachments to spindle microtubules (amphitely fixation), the SAC is inactivated, and chromosome segregation and cell division to proceed. If the chromosomes are not correctly attached to the spindle (erroneous attachments), kinetochores activate the SAC network, which inhibits the initiation of anaphase and preserves the cohesion of the sister chromatid [22,24,25]. Erroneous attachments include cases where only one kinetochore is attached to a spindle pole (monotely), both sister kinetochores are attached to the same pole (syntely), or one sister kinetochore is attached to both poles (merotely).

Furthermore, merotelic attachments are characterized by the absence of tension between sister kinetochores and are not detected by the SAC, and without correction, may result in chromosome mis-segregation due to slow chromatid migration speed [26,27]. Merotely is the primary mechanism of CIN in cancer cells [28]. In fact, it has been suggested that uncorrected merotelic attachments are the driving force behind the CIN phenotype observed in approximately 85% of all sporadic carcinomas [29].

### 2.2. The Role of CIN in Cancer Development and Progression

The role of CIN in the development of cancer is widely debated, since while some researchers consider that CIN is an early event in carcinogenesis that leads to the loss or inactivation of tumor suppressor genes [30,31,32], others postulate that CIN is a side effect of tumor growth, during which neoplastic cells lose and/or gain chromosomes relatively frequently [33]. In additon, it has been indicated that CIN facilitates the acquisition of mutations conferring aggressive or drug-resistant phenotypes during cancer evolution [34].

The impact of aneuploidy on gene expression implies that chromosomal copy number variation leads to an altered stoichiometry of proteins that interact physically or functionally. Stoichiometric perturbations of the protein interaction networks involved in chromosome segregation or the spindle assembly checkpoint can lead to errors in chromosomal segregation, aneuploidy and subsequent CIN [35].

In general, the chromosomal alterations that underlie CIN have emerged as prognostic markers for hematologic cancers and some solid tumors. In addition, the molecular characterization of cytogenetic alterations has provided important information on the mechanisms underlying tumorigenesis and on the treatments that target specific genetic abnormalities. Additionally, both CIN and heterogeneity have been associated with cancer progression, increased invasiveness, poor prognosis and, drug resistance [36,37,38,39,40], this is why some studies have given a clinical value to CIN in human cancers [39,40,41]. Furthermore, it has been reported that CIN is highest in the most aggressive and metastatic cancer types [42].

**Figure 3:**
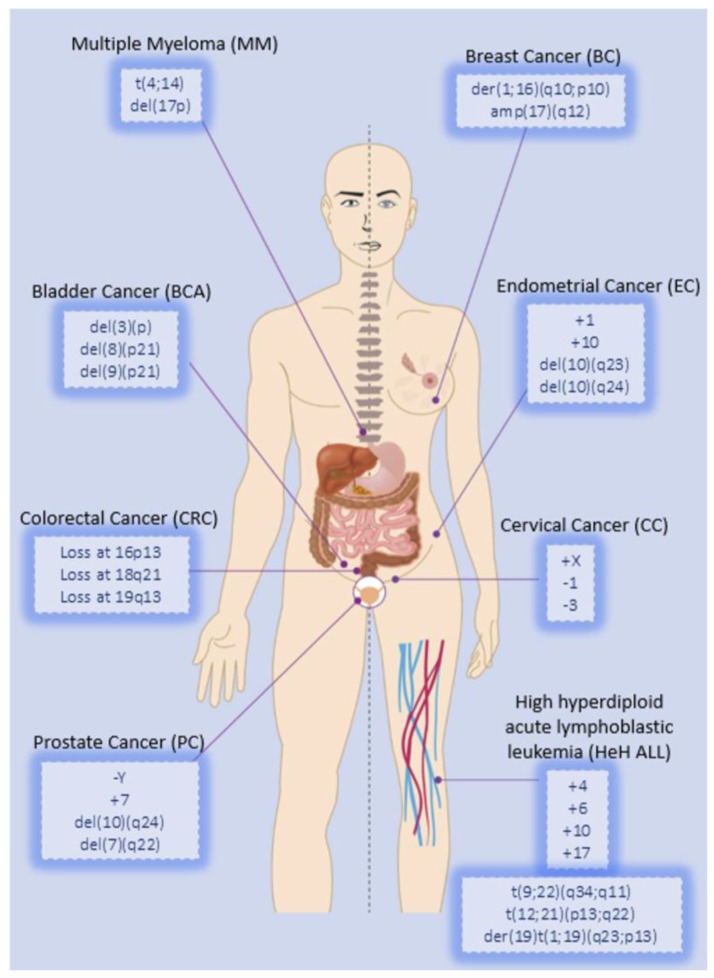
Chromosomal alterations most frequently observed in several types of cancer.

Considering that information regarding NCCAs is scarcely reported, here we indicate the CCAs most frequently observed in several types of cancer (these with higher incidence in the world population), and discuss their relationship with disease development and progression (Figure 3).

#### 2.2.1. Breast Cancer (BC)

BC is the second most common cancer in the world and the most frequent cancer among women, with an estimated 1.67 million new cases diagnosed in 2012 (25% of all cancers) [43]. BC is a heterogeneous disease, with appreciable patterns of chromosomal alterations. Kwei et al. (2010) [44] performed genomic profiling and postulated three different patterns of chromosomal alterations, which are differentiated by the frequency and complexity of such alterations. These patterns were called “simple”, “amplifier” and “complex”. The “simple” pattern is characterized by the presence of few gains or losses of whole chromosome arms. The “amplifying” pattern is characterized by the presence of focal high-level DNA amplifications, and the last pattern, the “complex”, is characterized by the presence of copy-number transitions and by numerous low-amplitude changes.

The “simple” pattern, exhibits few copy number alterations, with greater frequency of gains or losses of whole chromosome arms, most characteristically gain of 1q and 16p and loss of 16q. Additionally, a translocation resulting in a derivative chromosome der (1;16)(q10;p10), considered an early event in BC, has also been observed [45,46,47]. This pattern, also termed “simplex”, or “1q/16”, is primarily associated with estrogen receptor (ER)-positive, moderate to highly differentiated tumors, with luminal A gene-expression patterns and rarely observed in basal-like and HER2-related tumors [48,49]. Additionally, these alterations are observed in both early and invasive tumors. For instance, gain of 1q and loss of 16q are common in invasive carcinomas [50]. Loss of the long arm of chromosome 16 (16q) is also found in invasive ductal carcinomas, premalignant lesions [51] and in more than 60% of invasive lobular carcinomas. Although *CDH1* (E-cadherin) resides on 16q, to date, there is no evidence to show that loss of 16q in BC leads to the inactivation of this gene [44]. 

The “Amplifier” pattern is characterized by focal high-level DNA amplifications clustered on one or more chromosome arms. This pattern is associated with the luminal B and HER2-enriched subtypes [48,50]. Frequently amplified sites include 8p12 (*FGFR1*), 8q24 (*MYC*), 11q13 (*CCND1*), 12q15 (*MDM2*), 17q12 (*HER2*) and 20q13 (*ZNF217*). Some of these alterations have been noted to occur together [52], suggesting cooperating events and implying molecular subgroups. Amplified genes play important roles in signaling, cell proliferation, cell-cycle regulation and nucleic acid metabolism [50].

The third class of chromosomal alterations is characterized by a “complex” pattern of many gains and losses of low amplitude, which encompass short chromosomal regions. This pattern, called “complex”, results in a segmented profile with many variations of copy numbers, being more common in basal-like and triple-negative BC tumors. In spite of the complex patterns, the most frequent gains are observed in 10p, and the losses in 3p, 4p, 4q, 5q, 14q, 15q, and in some studies 17q [44].

Additionally, elevated aneuploidy is correlated with higher tumor grade, poorer survival and shorter times to recurrence and metastasis in most BC subtypes [53]. These observations suggest that CIN and the resultant alterations have important clinical implications that could be used not only to discriminate between different BC subtypes but also to direct therapy decisions. One of the most notable and classic examples is amplification of *HER2*, which occurs in approximately 15% of BC patients. This gene (also called *ERBB2*) is located on long arm of chromosome 17 (17q12) and encodes a transmembrane tyrosine kinase receptor, whose overexpression is a pharmacological target for the recombinant monoclonal antibody Trastuzumab (herceptin). In addition, the use of combination therapies, which include trastuzumab and chemotherapy, has been reported to reduce the rate of BC death in both the adjuvant and metastatic settings [54].

#### 2.2.2. Prostate Cancer

Prostate cancer (PC) is the second most common cancer in men. Worldwide, an estimated 1.1 million men were diagnosed with PC in 2012, accounting for 15% of all cancer diagnoses in men, with almost 70% of the cases (759,000) occurring in more developed countries [43].

There is a substantial body of literature that establishes the presence of CIN in PC, and various candidate chromosomes have been suggested to play a role in malignant development, including chromosomes 1, 7, 8, 10, 17 and X [55,56,57]. For instance, Al-Maghrabi et al. analyzed numerical CIN (aneuploidy) in PC patients and showed that gain of chromosome 8 was the most frequent change, followed by gain of chromosome 7 and chromosome Y aneusomy [58]. In addition, a strong correlation between chromosomal alterations and prognosis has been also established in PC. For example, tumors with 8q gains or more than two genetic copy number changes are correlated with poor outcomes. In fact, the prognostic significance of 8q gain in PC was recently reported [56].

Additionally, studies of peripheral blood lymphocytes (PBLs) from PC patients have also been performed. These studies found that the X-chromosome had a significantly higher mean level of spontaneous breaks in patients compared to those observed in controls. These results showed that spontaneous CIN in PBLs might be a potential biomarker for PC susceptibility [59]. In fact, an increased frequency of CIN in PBLs reflects the early biological effects of genotoxic carcinogens and individual cancer susceptibilities [60].

Furthermore, the metastatic potential of disseminated cell pools from metastatic PC patients was recently investigated. In this study, Holcomb et al. detected frequent losses in 8p23, 10q, 13q and 16q, and gains in 8q and Xq, alterations that are frequently identified in PC [61,62]. According to the authors, these results established the basis to elucidate the relationship between genomic alterations of disseminated tumor cells and progressive PC [61].

In addition, Baca et al. (2013) [63], by modeling the genesis of genomic rearrangements in PC, identified many DNA translocations and deletions that arise in a highly interdependent manner. This phenomenon was called “chromoplexy”, a term used to describe the complex genome restructuring. Such complex rearrangement events may disrupt tumor suppressor genes and creating oncogenic fusions in a coordinated way, possibly favoring tumor evolution not only in PC but in other neoplasms. The characterization of chromoplexia in cancer, which is indicative of structural CIN, could provide information on the initiation and progression of cancer, with broad implications for the detection, prevention and therapy of cancer. Together, these results suggest important implications for CIN in PC development, progression and evolution.

#### 2.2.3. Colorectal Cancer

Worldwide, Colorectal cancer (CRC) is the third most common cancer in men (746,000 cases, 10.0% of the total) and the second in women (614,000 cases, 9.2% of the total) [43]. CIN has been observed in 65% of CRC cases, lowest in stage 1 and highest in stage 4 disease [42]. Recurrent losses at 16p13 and 19q13, which are significantly associated with bad outcomes in stage 2 and 3 of the disease, have been observed in CRC [42]. Interestingly both regions co-occurred in the high-risk genetic instability groups. Additionally, allelic loss at chromosome 18q21 has been identified in the 70% of primary colorectal tumors, particularly in advanced-staged disease [64]. Tumor suppressor genes are localized within this region, including the gene *Deleted in Colorectal Carcinoma* (*DCC*), mutations of which are rarely detected in human colorectal tumors (6%) [65,66]. Additionally, *SMAD2* and *SMAD4*, which regulate cell growth, differentiation and apoptosis are also within this region; however, *SMAD2* and *SMAD4* mutations have been found in low frequency in CRCs [67,68].

Further studies found additional chromosomal alterations in CRC. For instance, Shih et al. analyzed 32 sporadic colorectal adenomas and identified a relatively high frequency of allelic imbalances on chromosomes 1p (10%), 5q (55%), 8p (19%), 15q (28%), and 18q (28%), with more than 90% of the adenomas showing allelic imbalance of at least one chromosomal arm [69]. In addition, CIN has been also reported in colorectal adenomatous polyposis. For instance, Cardoso et al. [70], for studying the aneuploid status of polyps from patients with germline *APC* or *MYH* mutations and found that among 60–80% of the polyps exhibited aneuploid changes, being the most frequent aberrations the losses of chromosomes 17p, 19q and 22q and the gains of chromosomes 7 and 13 [70]. These findings support the conclusion that chromosomal abnormalities can occur during the early stages of tumorigenesis. In fact, recent reports have indicated that brain metastases have higher frequency of gains and losses of whole chromosomes and generally more chromosomal aberrations than primary tumors [39,71].

Interestingly, it has also been reported that CIN in CRC can be a therapeutic target. For instance, Swanton et al. (2007) [72] observed that CIN-positive tumors are intrinsically resistant to taxanes due to the similarities between both: pathways that regulate the separation of chromosomes during mitosis, and pathways involved in taxanes responses. In fact, taxanes function primarily by interfering with spindle microtubule dynamics. When cells are exposed to conditions of prolonged mitotic stress in the presence of microtubule poisonous drugs, like taxanes, the SAC is eventually silenced and cells can exit mitosis [73].

#### 2.2.4. Cervical Cancer

Cervical cancer (CC) is the fourth most common cancer in women and the seventh most common overall, with an estimated 528,000 new cases in 2012 [43]. Despite treatment, distant metastasis and nodal recurrence will develop in approximately 30% of CC patients [74]. Numerical and structural chromosomal alterations, or a combination of the two, have been identified during the early stages of CC [75,76]. Structural and numerical chromosome 1 alterations are the most frequent karyotypic change in CC. Among the numerical chromosomal alterations, monosomies and polysomies of chromosomes 1, 3, and X are routinely used as positive genetic biomarkers to diagnose CC and predict the extent of disease progression [77,78]. It is also noteworthy that an increased frequency of spontaneous chromosomal aberrations was observed in patients with precancerous cervical lesions [79], indicating a possible role for CIN in CC progression. In fact, it has been suggested that aneuploidy status is a better prognostic predictor than lymph node status in CC [80].

In addition to the alterations indicated above, micronuclei (MN) have also been observed in CC. MN are extra-nuclear bodies that contain damaged chromosome fragments and/or whole chromosomes that were not incorporated into the nucleus after cell division [81]. MN are therefore the result of CIN. High frequency of MN has been reported in invasive CC, being suggested that the MN score of the exfoliated cervical cells, could be considered as an additional criterion to establish the risk of CC. However, due to the limited number of studies on MN scoring to assess CC risk [82] and on MN scoring in cervical pre-neoplastic and neoplastic conditions [76,83], their implications for CC have not been confirmed. It is noteworthy that according to recent reports, the presence of CIN may help distinguish patients with clinically significant cervical lesions from those who have insignificant lesions, thus discriminating the patient population [84].

#### 2.2.5. Endometrial Cancer

Endometrial cancer (EC) is a disease in which malignant cells form in endometrial tissues and is the leading cause of malignancy in the female genital tract, mostly affecting post-menopausal women [85]. Although the genetic alterations that underlie CIN in EC are poorly understood, a sequential accumulation of genetic alterations from benign to malignant primary lesions has been hypothesized; such alterations include a high frequency of chromosome 10q allelic deletions [86]. In particular, the regions 10q23 [87] and 10q25–q26 [88] have been strongly correlated with EC. Nevertheless, gains of chromosomes 1 and 10 represent the most common cytogenetic abnormality detected in EC [89]. For instance, Muresu et al. observed a high frequency of chromosome 1 and 10 trisomy/tetrasomy by analyzing archival tissues from a subset of 86 sporadic EC patients [90].

Interestingly, it has been indicated that CIN can also be found in PBLs from EC patients. The presence of CIN in PBLs is indicative of genome alterations, which are primarily characterized by imperfectly functioning DNA damage repair genes, including genes in the *MMR* family and cell cycle regulators such as *PTEN*, *PIK3*, *KRAS* and *BRAF* [91]. In fact, Bochkov et al. reported an increased level of spontaneous chromosomal aberrations in PBLs from EC patients compared with healthy women [92]. Similar results were recently reported by Nesina et al. who indicated that PBLs from most EC patients were characterized by genome destabilization, which was manifested by increased numbers of spontaneous and induced chromosomal damage, hypersensitivity to mutagenic factors, and hidden CIN [93]. Hidden CIN is defined as chromosomal alterations caused by mutagenic exposure to some exogenous or endogenous genotoxic factors [94], which are observed in low frequency (index lower than 1.0). In addition, according to Nessina et al. hidden CIN is one of the manifestations of human genomic instability induced by exposure to radiation, and is a sign of genome destabilization that likely plays a role in EC pathogenesis [35].

#### 2.2.6. Bladder Cancer

Bladder Cancer (BCA) is one of the most common cancers in the world. An estimated 430,000 BCA cases occurred in 2012, making the disease the ninth most common cause of cancer for both sexes combined [43]. Numerous, nonrandom chromosomal deletions have frequently been detected in BCA [95], including deletions of 3p, 8p, 9p, 11p, 11q and Y. Additionally, gain of 1q, 8q, 17q and 20q have also been found [96,97,98,99]. Furthermore, specific chromosomal deletions are associated with BCA progression, and such progression correlates with specific stages of tumor development.

Deletions on chromosome 3p in BCA focused the attention of many researchers, because studies in other types of cancer suggested the presence of tumor suppressor genes in this chromosomal region. For instance, deletions on chromosome 3p have been associated with invasive tumors and have been found in approximately 25% of BCA cases [100,101]. Chromosome 8 deletions, which most frequently affect the region 8p21–22 [102,103], have been observed in 25–50% of BCA cases [95] and have been significantly correlated with cancer progression [55,103]. Deletions and losses of chromosome 9 have been reported as the most frequently observed in BCA. Loss of chromosome 9 is the only type of chromosome loss identified at the early tumor stages T_0_ and T_1_ [95], while at later stages loss of other chromosomes, concomitantly with the loss of chromosome 9, were detected [95]. Considering the above, it has been indicated that the total loss of chromosome 9 represents an initial event in the formation of bladder tumors [104]. Deletions of chromosome 9 lead to the loss of genes that encode proteins that activate *Rb* and *p53*, important tumor suppressors [95]. Chromosome 11 deletions are seen in BCA at a high frequency (71.43%) [105], while loss of chromosome 17 has been found in 60% BCA cases and has been associated with advanced disease [95].

The implications of CIN in BCA have reached great importance in recent years, such that clinical tests have been developed specifically to evaluate genomic instability as a molecular marker for the early detection of BCA. In fact, the international consensus panel on bladder tumor markers, recommended a multicolor fluorescence in situ hybridization assay to detect copy number variations of chromosomes 3, 7 and 17, and at the 9p21 locus in exfoliated urothelial cells [106]. This test has shown reasonable performance in detecting BCA in male chemical workers with previous exposure to aromatic amines [107].

#### 2.2.7. Multiple Myeloma

Multiple Myeloma (MM) constituted 0.8% of all cancers worldwide (114,000 new cases in 2012) [43]. MM is a cancer formed by malignant plasma cells. Normal plasma cells are found in the bone marrow and are an important part of the immune system. This neoplasia is characterized by the high frequency and consequent accumulation of chromosomal alterations [108]. Furthermore, the complexities of the genomic alterations characteristic of this neoplasm have been correlated with different grades of CIN. Among these alterations, individual abnormalities such as t(4:14) [109] and the deletion of the short arm of chromosome 17 (del(17p)) [110] are associated with poor outcomes in several treatment contexts [111,112]. Additionally, 17p deletions are also correlated with poor prognosis in MM patients treated with conventional and thalidomide-based chemotherapies [110]. These observations strongly implicated CIN as an important biological and prognostic marker in MM [113].

#### 2.2.8. High Hyperdiploid Acute Lymphoblastic Leukemia (HeH ALL)

In childhood B-cell precursor acute lymphoblastic leukemia (ALL), the most common cytogenetic abnormality is the high hyperdiploidy (51–67 chromosomes), which occurs in 25–30% of all pediatric B-cell precursor ALL. Of note that high hyperdiploidy has been strongly associated with childhood ALL, since modal numbers of 51–67 have been observed in low frequency in adult B-lineage ALL and rarely in T-cells or in Burkitt's leukemia/lymphoma [114].

HeH ALL is cytogenetically characterized for nonrandom gains of chromosomes X, 4, 6, 10, 14, 17, 18, and 21 [114,115]. Gains of chromosome 21 are the most frequent numerical alterations, showing between three or more copies in 90–100% of cases [116,117]. In addition to the chromosomal gains, approximately 50% of HeH ALL cases have also structural chromosome aberrations [118,119]. The structural chromosomal alterations observed at high frequencies in HeH ALL are indicated in Table 1 [82,120].

Interestingly, previously published data suggested a cell-to-cell variation in HeH ALL at diagnosis [121,122]; however, further results did not verify this indication. Nevertheless, additional research brought further evidence of a high level of CIN for chromosomes 4, 6, 10, and 17 in HeH ALL patients at initial presentation [123]. In fact, Talamo et al. reported that CIN values in HeH ALL patients were higher than those in the negative control group, which would corroborate the potential role of CIN in HeH ALL pathogenesis [123].

Regarding the associations of CIN with outcomes, it has been indicated that whereas trisomies of chromosomes 4 and 6 did not affect prognosis, concurrent trisomies of chromosomes 10 and 17 were associated with better outcomes, and trisomy of chromosome 5 was associated with a poor prognosis [124]. Additionally, Moorman et al. found an association between trisomies of chromosomes 4, 10, and 18 and improved outcomes, but only trisomies of chromosomes 4 and 18 had an independent impact in multivariate analysis [119]. Considering these conflicting results, it is important to highlight the need to carry out additional studies to determine whether CIN is a general feature of HeH ALL and to what extent it affects outcomes, as this would be useful information for therapy decisions.

## 3. The Role of CIN in Anticancer Therapy Responses

The importance of CIN in therapeutic responses results from the fact that chromosomal alterations can lead to altered gene regulatory interactions and varying protein concentrations, both of which could impact cellular responses to treatment [125]. In this regard, it has been indicated that CIN leads to heterogeneous gene expression within a tumor, which could favor the emergence of drug-resistant cell populations, promoting survival in a fraction of tumor cells [126]. However, while some studies have associated high CIN with poor patient outcomes and drug resistance [127], others have indicated that it is associated with better responses [4,128]. In fact, it has been indicated that targeting CIN for cancer therapy can induce genome chaos, which contributes to an increased CIN and therefore to the possible acquisition of proliferative advantages and resistance to therapy [129,130].

### 3.1. Therapeutic Strategies Based on CIN

**Figure 4:**
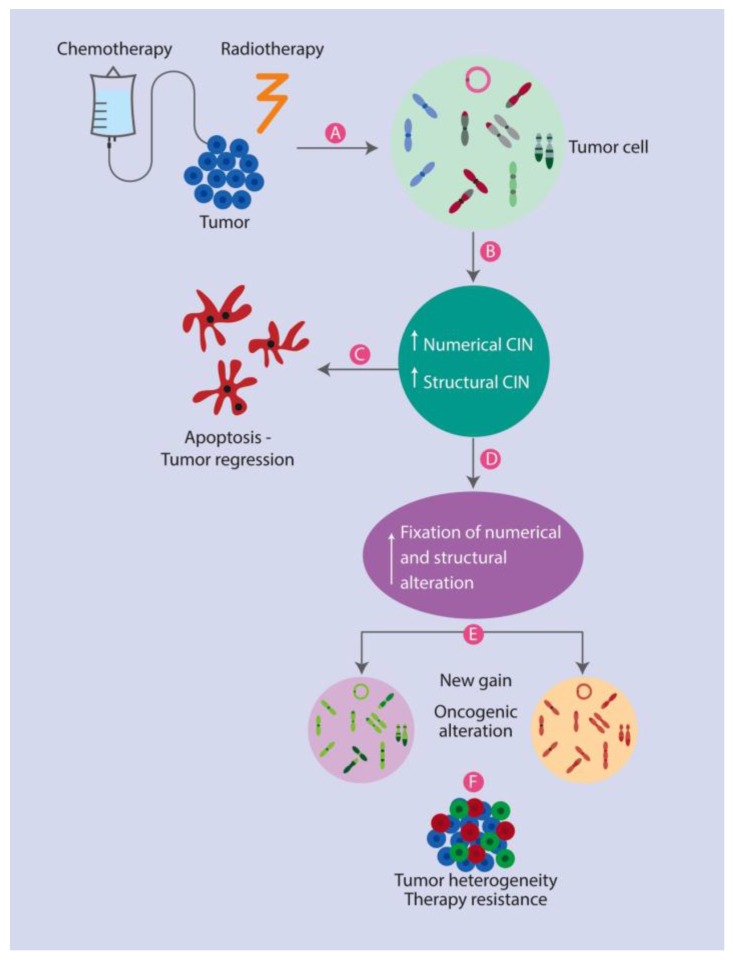
CIN Positive or Negative Response to Treatment. (**A**) The administration of chemotherapy or radiotherapy to tumor cells (**B**) can lead to the induction of new numerical and structural chromosomal alterations; This condition could generate two cellular responses; (**C**) one of them related to the induction of apoptosis (probably due to the excess of genotoxicity), improving the prognosis for the patient by tumor regression, and the other (**D**) related to the fixation of numerical and structural alterations; (**E**) consequently leading to clonal expansion of new oncogenic alterations and thus (**F**) to an overall increase in heterogeneity and development of resistance to therapy.

Before considering CIN as a therapeutic strategy, it must be detected and monitored to know if it can be used as a tool to predict tumor phenotypes, and in this way, contributes to establishing personalized treatment [35,128,131]. This is made possible by determining if CIN makes a tumor more adaptable and better prepared to evolve towards developing resistance to a treatment, or on the contrary, it allows regression of the tumor through cellular collapse [4,34] that leads aberrant cells to undergo apoptosis (Figure 4).

FISH is the most commonly used method to evaluate CIN in patient samples [127,132,133,134], and studies carried out with this technique in parallel with other findings have shown that increased CIN can positively or negatively impact treatment responses [6,135].

### 3.2. The Association between CIN and Poor Prognoses

CIN is generally correlated with tumor development, and innumerable studies have suggested that the aneuploidy that arises as a consequence of CIN in solid tumors favors tumor progression and metastasis [4,5,127,136,137]. It has been demonstrated in several cancer types that CIN-mediated intra-tumoral variability is associated with increased disease aggressiveness, a phenomenon that arises as a consequence of tumor heterogeneity, or the presence of multiple cell clones at the genetic level, which makes the tumor more adaptable and better prepared to evolve resistance [4,5]. For example, studies of ER-positive BC patients [135] and women with ovarian cancer [128] have shown increased CIN in women with resistant disease. In cases where increased CIN contributed to tumor development, therapeutic strategies aimed at decreasing its rate, and thereby inhibiting the processes that led to poor chromosomal segregation or structural changes in cancer cells, have been applied [4].

### 3.3. CIN and Its Potential Beneficial Effects for Therapy

Without a doubt, the role of the CIN in tumor development is a subject that is currently being debated, and contradictory, results generated from animal models show that CIN is poorly tolerated by cancer cells [6].

It has been indicated that although CIN can be beneficial for tumors by providing advantageous alterations, it can also generate vulnerabilities that can be exploited therapeutically. In fact, CIN can generate “synthetic lethal” interactions specifically in tumor cells, by inducing gene dependencies not present in normal cells [138]. For instance, in BC, *BRCA1* and *BRCA2* deficiency leads to a marked sensitivity to poly(ADP-ribose) polymerase (PARP) inhibitors [139,140]. PARP plays an important role in the repair of single-strand breaks, and it is believed that its inhibition leads to the collapse of the replication fork and double-strand breaks, which for their repair depend on homologous recombination. Notably, because *BRCA* carriers are only fully deficient for BRCA function their tumors (not in normal tissues), PARP inhibitors are likely to be highly tumor-specific [44].

Birkbak et al. 2011 [6] demonstrated that the extreme CIN in ER negative breast cancer tumors was associated with best prognosis, and similar results have been also observed in ovarian, gastric and non-small cell lung cancer. The therapeutic strategy in cases where CIN generates cell death aims to exacerbate this condition in order to induce tumor cell death [19,33,141,142,143].

The experimental evidence showing that the increase in CIN in some tumors triggers its reduction has been based mainly on animal models, in which, when treating mice by chemical carcinogens, in order to induce high levels of CIN, culminate in tumor cells death and consequently in reduction or destruction of the tumor, a phenomenon that can be understood as a better prognosis [144,145]. The analysis of these results suggests a possible explanation of how exacerbated CIN could be operating against the tumor: too much CIN leads to excessive mutations that result in the loss of benefits that the cells had initially acquired toward their tumor transformation [146,147].

## 4. CIN in Naturally Occurring Congenital Aneuploidy of Non-Cancerous Origin

CIN plays important roles not only in neoplasia but also in other disease types. Although in humans, whole chromosome aneuploidies are fatal, some of them may be viable but cause congenital diseases: trisomy 13 (Patau syndrome), trisomy 18 (Edwards syndrome), trisomy 21 (Down syndrome), and monosomy X (Turner syndrome). However, it has been reported that the increased rates of CIN observed in these syndromes may increase the risk of developing certain types of cancer. For instance, children with Down syndrome have a high risk of acute myeloid leukemia [148]; Edwards syndrome patients are at risk of developing Wilms’ tumor; women with Turner syndrome have an increased risk of gonadoblastoma and childhood brain tumors [149]; men with Klinefelter syndrome have elevated risks of lung cancer, BC and non-Hodgkin’s lymphoma [150], while men with Y polysomy have reported rates of cancer incidence similar to those observed in the general population [151]. Interestingly, although few studies have explored CIN in human trisomies, data reported to date, suggest that cells from Down, Edward, Turner and Patau syndrome patients may be karyotypically more unstable than cells from normal diploid individuals.

In addition to above syndromes, CIN has also been observed in Mosaic variegated aneuploidy (MVA) syndrome. This syndrome is a rare disorder in which some cells in the body have an abnormal number of chromosomes instead of the usual 46 chromosomes (aneuploidy). Among the aneuploidies most commonly observed in this syndrome are the monosomies and the trisomies. 

MVA syndrome can be caused by mutations in the *BUB1B* gene or the *CEP57* gene. Both genes play very important functions in the process of cell division, since they encoding proteins involved in mitotic spindle checkpoint and in microtubule stabilization, respectively [152]. MVA syndrome is characterized by multiple mosaic aneuploidies, and a distinct phenotype [153]. Other common characteristic of MVA syndrome is the increased risk of cancer. Cancers that occur most frequently in affected individuals include rhabdomyosarcoma (a cancer of muscle tissue), Wilms tumor (a form of kidney cancer) and leukemia [154,155]. The high incidence of tumors in MVA patients suggests a causal link between CIN and tumor formation [33]. All these results suggested that CIN could contribute to the development of cancer in naturally occurring congenital aneuploidy of non-Cancerous Origin.

## 5. Conclusions

The tumor-promoting role of CIN has been widely reported in several neoplasms; however, although our understanding of CIN has increased in last years, it is still necessary to consider its consequences in the context of cancer as a heterogeneous and complex disease, instead of one in which CIN only contributes to tumor progression in a simple and autonomous way. In fact, the studies performed to date suggest an important role for CIN in both the outcome and in the responses and resistance to therapy. Thus, CIN is an important target to be considered as we develop novel and more effective anticancer treatments. Furthermore, given that cancer is characterized by unstable and chaotic karyotypes, and that such CIN is mainly defined by the presence of NCCAs, identifying and reporting such alterations is clinically relevant. Furthermore, considering that NCCAs are a source of genetic variation not previously recognized, their identification could contribute not only to increase our knowledge about cancer but also to identify new therapeutic opportunities.

## Figures and Tables

**Table 1:** Chromosomal alterations observed at high frequencies in High Hyperdiploid Acute Lymphoblastic Leukemia (HeH ALL).

Structural Chromosomal Alterations	Number of Cases
t(9;22)(q34;q11)	991
t(12;21)(p13;q22)	367
der(19)t(1;19)(q23;p13)	263
i(9)(q10)	183
i(17)(q10)	158
i(7)(q10)	155
t(11;19)(q23;p13)	138
del(9)(p21)	134
del(12)(p12)	123
del(11)(q23)	114
del(12)(p13)	77
i(21)(q10)	68
add(19)(p13)	60
dic(9;20)(p11;q11)	52
dic(9;20)(p13;q11)	50
